# Mediterranean Diet Reduces Social Isolation and Anxiety in Adult Female Nonhuman Primates

**DOI:** 10.3390/nu14142852

**Published:** 2022-07-12

**Authors:** Corbin S. C. Johnson, Brett M. Frye, Thomas C. Register, Noah Snyder-Mackler, Carol A. Shively

**Affiliations:** 1 Department of Psychology, University of Washington, Seattle, WA 98105, USA; cscjohns@uw.edu (C.S.C.J.); nsnyderm@asu.edu (N.S.-M.); 2 Department of Pathology, Section on Comparative Medicine, Wake Forest School of Medicine, Winston-Salem, NC 27157, USA; bmfrye@wakehealth.edu (B.M.F.); register@wakehealth.edu (T.C.R.); 3 Department of Biology, Emory and Henry College, Emory, VA 24327, USA; 4 Center for Studies in Demography and Ecology, University of Washington, Seattle, WA 98105, USA; 5 Center for Evolution & Medicine, Arizona State University, Tempe, AZ 85281, USA; 6 School of Life Sciences, Arizona State University, Tempe, AZ 85281, USA; 7 School for Human Evolution and Social Change, Arizona State University, Tempe, AZ 85281, USA

**Keywords:** Mediterranean diet, Western diet, nonhuman primates, social isolation, anxiety

## Abstract

Dietary composition is associated with the differential prevalence of psychiatric disorders; the Western diet confers increased risk, while the Mediterranean diet appears to reduce risk. In nonhuman primates, anxiety-like behaviors and social isolation have been linked to both Western diet consumption and increased inflammatory disease risk, and recent evidence suggests that diet composition may affect immune system function in part through its effects on behavior. This is particularly important in the context of the global COVID-19 pandemic in which social isolation has been associated with disease. Here, we examined the effects of Western- and Mediterranean-like diets on social behavior in a randomized, 34-month preclinical trial in middle-aged female cynomolgus macaques (*Macaca fascicularis*). Diet induced rapid and persistent changes in a suite of behaviors. After just three months of experimental diet consumption, a composite measure of diet-altered behavior (DAB) significantly differed between the two diets (*p* = 0.014) and remained different throughout the 24-month experimental observation period (*p* = 2.2 × 10^−8^). Monkeys fed the Western diet spent more time alone (FDR = 4.4 × 10^−5^) and displayed more anxiety behavior (FDR = 0.048), whereas monkeys fed the Mediterranean diet spent more time resting (FDR = 0.0013), attentive (FDR = 0.017), and in body contact with groupmates (FDR = 4.1 × 10^−8^). These differences were largely due to changes in behavior of animals fed the Mediterranean diet, while Western-diet-fed-animals exhibited similar behaviors compared to the eight-month baseline period, during which all monkeys consumed a common laboratory diet. These observations provide experimental support in a nonhuman primate model, demonstrating a potential therapeutic benefit of the Mediterranean diet consumption to reduce social isolation and anxiety and thus mitigate social isolation-associated disorders that often accompany illness and disability.

## 1. Introduction

Long-standing evidence supports an association between diet composition and the incidence of psychiatric disorders. Observational studies have demonstrated an increased prevalence of anxiety in people self-reporting consumption of a Western diet, which is rich in simple sugars and animal sources of saturated fats and proteins [1,2,3], and decreased prevalence in those self-reporting adherence to a Mediterranean diet, rich in plant sources of mono/polyunsaturated fats, proteins, and antioxidants [4,5,6]. While psychiatric disorders have an independent and profound impact on human health, they are often comorbid with cardiovascular [7,8,9,10,11] and inflammatory diseases [12,13], suggesting a shared underlying pathology. In support of this, a large body of evidence across taxa has supported a physiological response to social adversity (e.g., adverse events early in life, low social status, and social isolation) that is linked to reduced health span and lifespan [14]. For instance, in both human and nonhuman primates (NHPs), individuals that are more socially integrated have longer, healthier lives than their more socially isolated counterparts [15,16,17,18,19,20,21,22]. Thus, understanding the effects of common human dietary patterns on socioemotional behavior, in particular social isolation and loneliness, may elucidate neurobiological pathways through which diet impacts human health.

Long-term effects of diet composition on human behavior are difficult to ascertain as lengthy feeding trials are expensive, and self-reported food intake data are unreliable [23,24]. Similarly, long-term assessments of socio-behavioral variables in humans are most often based on self-report [25,26], confounding perceived with actual events. In addition, definitions of social isolation may vary across studies [27], and social isolation (objective physical separation from social contacts) and loneliness (distress from perceived social isolation) are often conflated in the literature [19]. Macaques have long been used as models for human health and disease as they share many core genetic, physiological, and behavioral phenotypes, including omnivory, with humans [28,29,30,31,32,33]. Like humans, individual NHPs may vary in their degree of social integration and isolation, and multiple factors may influence sociality, including age, sex, social status, kin networks, and familiarity with social partners [34]. These similarities suggest that NHPs may share mechanisms linking social isolation and health with humans, and direct observation of social isolation avoids the confounds inherent in human studies, thus supporting their use as a model system. Currently, little is known about how diet impacts social isolation and whether other measures of the social environment, like social status, can affect those relationships.

To address these questions, we drew on data from our randomized preclinical trial in socially housed cynomolgus macaques (*Macaca fascicularis*). The monkeys first consumed the lab-standard monkey chow during an eight-month baseline phase prior to assignment to groups that were fed either a Mediterranean or a Western diet, formulated to mimic human diet patterns, for 26 months [35]. Our previous findings from this preclinical trial demonstrated that relative to the Mediterranean diet, the Western diet increased body weight, body fat, insulin resistance, and hepatosteatosis [35]; altered gut microbiome composition [36]; exacerbated autonomic and hypothalamic–pituitary–adrenal responses to psychosocial stress [37]; altered brain neuroanatomy [38]; and drove inflammatory polarization of circulating immune cells [39]. In the latter article, we reported that the second axis of variance in a principal component analysis of behavior during the first 14 months of the study was strongly associated with diet [39]. High scores on this “diet-altered behavior” principal component were positively associated with Mediterranean diet consumption, time spent in body contact, and time resting. Low scores were associated with Western diet consumption, time spent alone, anxiety behaviors, and proinflammatory immune cell gene expression. This behavioral phenotype was remarkably similar to that seen in the juvenile offspring of Japanese macaques that consumed a Western diet in the perinatal period [40] and is concordant with a human study in which the Western diet was a risk factor for loneliness [41].

Here we present an in-depth analysis of long-term changes in socioemotional behavior that differed between those that consumed a Western versus a Mediterranean diet pattern. We demonstrate the relatively rapid appearance of diet-altered behavior after just three months of experimental diet consumption and the persistence of diet-distinct patterns of socioemotional behavior throughout the entire 24 months of observation. We show that diet induces changes in affiliation, activity, and anxiety. These behavioral differences between the two diet groups were driven largely by changes in behavior from the baseline phase in the Mediterranean-fed monkeys.

## 2. Methods

### 2.1. Subjects

Adult (age: mean = 9.0, range = 8.2–10.4 years, estimated by dentition), female cynomolgus macaques (*Macaca fascicularis*) were socially housed in groups of 3–4 in 3 m × 3 m × 3 m enclosures, with daylight exposure, on a 12/12 light/dark cycle with monkey chow (Table 1) and water available ad libitum during an eight-month baseline phase. Social groups were then randomized to receive either the Western-like (hereafter “Western;” *n* = 5 groups, *n* = 21 monkeys) or Mediterranean-like (hereafter “Mediterranean;” *n* = 6 groups, *n* = 17 monkeys) diet. The two diet groups were balanced on markers of overall health measured during the baseline phase, including body weight, body mass index, circulating basal cortisol, total plasma cholesterol, and plasma triglyceride concentrations [35]. All animal manipulations were performed according to state and federal laws and guidelines from the US Department of Health and Human Services and the Animal Care and Use Committee of Wake Forest School of Medicine.

### 2.2. Experimental Diets

Diet composition and feeding have been described in detail [35]. Following the eight-month baseline period, macaques consumed one of two experimental diets formulated to match human Western and Mediterranean dietary patterns for 26 months. The experimental diets were isocaloric with respect to macronutrients and identical in cholesterol content (~320 mg/2000 kilocalories per day) but differed in composition. The Western diet was designed to mimic the diet typically consumed by middle-aged American women [42], resulting in a diet rich in saturated fats, sodium, and refined sugars with fats and proteins mostly from animal sources [45]. In contrast, the Mediterranean diet was formulated to reflect the human Mediterranean diet in high levels of monounsaturated fats and a lower omega-6:omega-3 fatty acid ratio than the Western diet, with protein and fat, derived mostly from plant sources [46]. Key Mediterranean ingredients included English walnut powder and extra-virgin olive oil, which were provided to participants in the Prevención con Dieta Mediterránea (PREDIMED) primary prevention trial [48]. The macronutrient composition of the experimental diets compared to monkey chow and human diet patterns are shown in Table 1. The composition of experimental diets has previously been published [35] and is described in Supplementary Table S1. Diet preparation is described in Supplementary Note S1. Monkeys were provided a 120 kcal diet per kg of bodyweight per day (120 kcal/kg/day), which was enough so that 10% of the diet was left at the end of the day, ensuring all group members had adequate access. There were no differences in caloric consumption as previously described [35].

### 2.3. Behavioral Characterization

Behavioral data were collected weekly during two 10-min focal observations [49], balanced for the time of day for six consecutive weeks during months 3–4 of the baseline phase (2 h/monkey total) and for 24 consecutive months beginning the third month of the treatment phase (approximately 200 behavior samples/monkey, mean = 31.0 h/monkey, and 1178 observation hours total). Inter-rater reliability was maintained at ≥93%, and no other research activities were ongoing during behavior observations. Behavioral data were not collected if the animal was ill or temporarily removed from the social group for evaluation. Thus, the length of observation varied from 0–80 min/monkey/month (median = 80 min/monkey/month). Data were collected on 38 animals, 21 in the Western group and 17 in the Mediterranean group (two Mediterranean animals had zero observations for one month). All recorded behaviors are operationally defined in Supplementary Table S2. Briefly, these behaviors included the frequency of aggressive and submissive behaviors differentiated by severity and directionality of behaviors; time spent in close proximity, alone, or in body contact with group mates; time spent giving or receiving grooming; time spent attending to the environment (attentive, investigating, or fearfully scanning); activity (time spent lying down with eyes open, resting with eyes closed, or in locomotion); and the frequency of anxious behavior defined as self-grooming and scratching [50,51,52,53,54]. Behaviors were combined into summary behaviors (e.g., “mild aggression”, defined as the sum of noncontact aggression events including display, displace, and threat), as previously described [30]. The outcomes of agonistic interactions were also tabulated monthly to determine social status. Based on these win-loss interactions, females were assigned a relative dominance rank, which represented the proportion of females in their group that was submissive to them. Thus, the highest-ranking female was given a relative rank value of 1, and the lowest-ranking female a relative rank value of 0. Relative ranks were stable over the experiment and significantly correlated between the baseline and treatment phases (Spearman’s rho = 0.90, *p* < 0.0001) [37]. Thus, the mean relative ranks were calculated for the duration of the experiment, and animals were classified as dominant if they had a mean relative rank >0.5 and subordinate if their mean relative rank was ≤0.5.

### 2.4. Statistical Analyses

The baseline and monthly treatment phase behavioral data were scaled, and principal component analysis was conducted using the PCA function of the R package FactoMineR [39,55]. Similar to our previous report, the first principal component of behavior accounted for 59% of the variance and was related to relative rank but not diet. The second principal component was significantly associated with diet and accounted for 19% of the variance and was used to calculate Diet-Altered Behavior (DAB) Scores [39]. Individual DAB scores were then calculated for each monkey for each month as a linear combination of the observed incidence of each behavior scaled and multiplied by the loading of each variable onto the second principal component (PC2, diet-altered behavior or DAB) weighted by the eigenvalue of PC2. This approach incorporated all 20 behaviors for which we collected data, but weighted behaviors by their contribution to the DAB score axis, such that time in body contact and resting had strong positive loading scores, while time alone and rate of anxiety behaviors had strong negative loading scores, and time in close proximity and grooming contributed slightly to an individual’s DAB score.

We tested baseline differences between the two groups in each behavior or DAB scores using Welch–Satterthwaite *t*-tests. Analyses of variance or covariance were used to test the relationship between diet and behavior, where the behavior at baseline was included as a covariate in the analysis of covariance (ANCOVA) if it was a significant predictor of the treatment phase behavior (*p* < 0.05); otherwise, ANOVA was used. ANCOVA was used to test the a priori hypothesis that the DAB score changed in the first month of behavioral observations (third month of the treatment phase). A repeated measure ANCOVA was used to test for the stability of the DAB score over time. Following this, ANCOVA was used as a post-hoc test to determine in which months the DAB scores of the two diet groups were significantly different. The *p*-values for the main effects of diet of each month were corrected for multiple hypothesis testing using the qvalue false discovery rate function from the qvalue R package [56] and reported as the false discovery rate (FDR). Analysis of variance was used in the same manner to test for differences in each of the 20 behaviors collected.

## 3. Results

We previously reported a significant difference between diet-altered behavior (DAB) scores of the two diet groups at 14 months of experimental diet consumption and examined the association between the DAB score and monocyte transcripts of immune function [39]. Here, we evaluated how quickly the DAB score diverged between the diet groups and how long the effect persisted, and we examined the underlying differences in individual behavior.

### 3.1. Diet Groups Did Not Significantly Differ in Any Behaviors during the Baseline Phase

**Figure 1:**
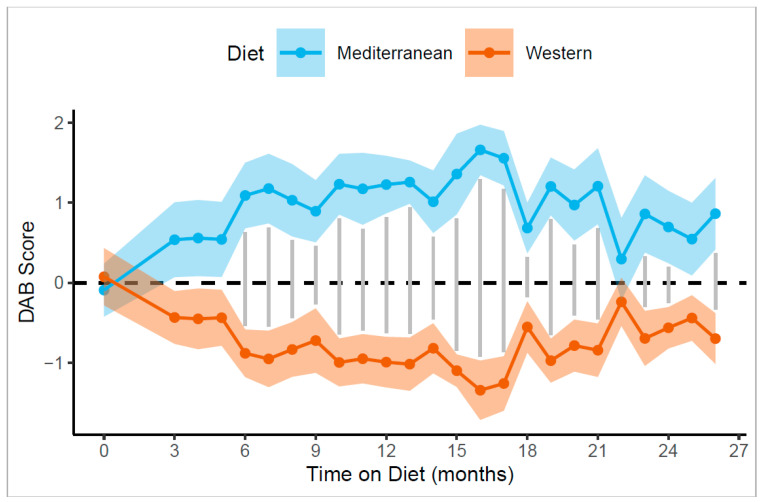
Diet-induced rapid changes in the DAB scores that persisted throughout the experiment. Monkeys consuming the Mediterranean diets (blue points [mean] and ribbons [SEM]) exhibited higher DAB scores in the first month of behavioral observation (three months on experimental diets; F _[1,35]_ = 6.7, *p* = 0.014), as well as consistently higher DAB scores during the treatment phase than did monkeys consuming the Western diets (orange points [mean] and ribbons [SEM]; F _[1,33]_ = 53.3, *p* = 2.2 × 10^−8^). There was a significant interaction between month and diet (F _[10.3,341.2]_ = 2.2, *p* = 0.019). Holm–Bonferroni-adjusted post-hoc analyses demonstrated significant differences in 19/24 months at *p* < 0.05, as indicated by vertical lines between points for each month, and an additional 4/24 months at *p* < 0.10.

There was no significant difference in the DAB score between the two treatment groups during the baseline phase during which the monkeys consumed a standard “monkey chow” diet (t_(36.0)_ = −0.3, *p* = 0.73; Figure 1, Supplementary Table S3). There were no significant differences between the diet groups during the baseline phase in any of the twenty individual behaviors measured (all *p* > 0.05; see Supplementary Table S2 for a list of behaviors measured and their operational definitions).

### 3.2. Diet-Induced Changes in Activity, Affiliation, and Anxiety

In contrast with the baseline phase of the experiment, we observed differences in DAB scores between the two diet groups in the experimental phase. Over the 24 months in which monkeys were fed experimental diets, the Mediterranean group had significantly higher DAB scores than the Western group (x_Mediterranean_ = 0.98, x_Western_ = −0.79; ANCOVA F _[1,33]_ = 53.3, *p* = 2.2 × 10^−8^; Figure 1).

**Figure 2:**
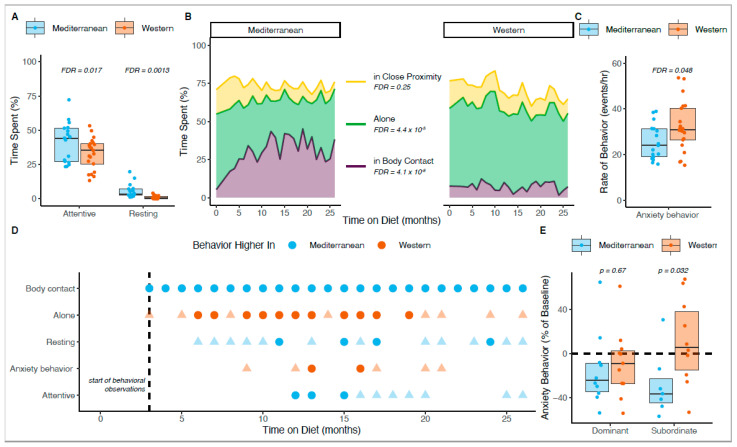
Changes in behavior following experimental diet treatments. (**A**) Monkeys in the Mediterranean group (blue boxes and points) spent more time attentive (ANCOVA F _[1,35]_ = 8.6, FDR = 0.017) and resting (AVOVA F _[1,36]_ = 15.6, FDR = 0.0013) than those in the Western group (orange boxes and points). (**B**) The left plot shows how monkeys in the Mediterranean group spent time in proximity to one another, while the right plot shows the same for monkeys in the Western group. Monkeys in the Mediterranean group spent more time in body contact (ANCOVA F _[1,35]_ = 60.8, FDR = 4.1 × 10^−8^; purple) and less time alone (ANCOVA F _[1,35]_ = 27.4, FDR = 4.4 × 10^−5^; green) than the Western group, while there was no significant difference in time spent in close proximity (ANCOVA F _[1,35]_ = 0.8, FDR = 0.25; yellow). (**C**) The rate of anxiety behaviors observed in the Western group was significantly higher than in the Mediterranean group during the treatment phase (ANCOVA F _[1,35]_ = 5.9, FDR = 0.048). (**D**) Points indicate months in which there was a significant difference between diet groups for a particular behavior. Circular points are significant after correcting for the multiple hypothesis test, whereas triangular points are those that do not pass the heightened threshold. Blue (Mediterranean) or orange (Western) points indicate which group displayed more of the behavior for that month. (**E**) Dominant monkeys in both diet groups decreased anxiety from baseline (left), although there was no significant difference in change in anxiety between diet groups (t_(18.3)_ = 0.67). Only subordinates fed the Mediterranean diet decreased from baseline (right). Within subordinate animals, anxiety decreased significantly more in the Mediterranean group than in the Western group (t_(14.9)_ = 2.37, *p* = 0.032).

We next determined which behaviors responded to diet. Of the twenty behaviors measured, five (resting, body contact, attentive, anxiety, alone) were significantly different between diet groups during the treatment phase (Figure 2A–C). The Mediterranean group spent significantly more time resting (x_Mediterranean_ = 5.7%, x_Western_ = 1.2%; ANOVA F _[1,36]_ = 15.6, FDR = 0.0013; Figure 2A), and were more attentive than the Western group (x_Mediterranean_ = 41.4% of attentive time, x_Western_ = 33.0%; ANCOVA F _[1,35]_ = 8.6, FDR = 0.017; Figure 2A).

Time spent in body contact and proximity to groupmates (i.e., within arm’s reach) in contrast to alone, are measures of social integration or affiliation. The Mediterranean group spent significantly more time in body contact than the Western group (x_Mediterranean_ = 31.6%, x_Western_ = 7.3%; ANCOVA F _[1,35]_ = 60.8, FDR = 4.1 × 10^−8^; Figure 2B), whereas the Western group spent more time alone (out of arm’s reach of others) than the Mediterranean group (x_Mediterranean_ = 32.4%, x_Western_ = 51.4%; ANCOVA F _[1,35]_ = 27.4, FDR = 4.4 × 10^−5^; Figure 2B). There was no significant difference in time spent in close proximity (within arm’s reach) between the two diet groups (x_Mediterranean_ = 9.6%, x_Western_ = 11.6%; ANCOVA F _[1,35]_ = 0.8, FDR = 0.25; Figure 2B), suggesting that monkeys in the Mediterranean group were trading time alone for time in body contact, and shifting towards a more affiliative phenotype.

The Western group displayed more anxiety behavior than the Mediterranean group (x_Mediterranean_ = 25.4 events/h, x_Western_ = 32.2 events/h; ANCOVA F _[1,35]_ = 5.9, FDR = 0.048; Figure 2C). When taken together, these changes indicate that diet composition altered monkeys’ physical activity, affiliation, and anxiety.

### 3.3. Diet-Induced Changes in DAB and Affiliation Were Rapid

We compared the DAB scores and the five significantly altered behaviors between diet groups for the first month of behavioral data collection to understand how quickly behavior changed in response to experimental diets. After one month of behavior observation (three months of experimental diet consumption), the DAB scores of Mediterranean-fed monkeys were significantly higher than those of Western-fed monkeys (x_Mediterranean_ = 0.54, x_Western_ = −0.43; ANCOVA F _[1,35]_ = 6.7, *p* = 0.014; Figure 1), controlling for baseline DAB scores.

Of the behaviors that were significantly different throughout the experimental phase, only the changes in affiliation showed rapid changes. The Mediterranean group spent significantly less time alone (x_Mediterranean_ = 41.0%, x_Western_ = 58.7%; ANCOVA F _[1,35]_ = 8.14, Holm–Bonferroni adjusted *p* (*p_HB_*) = 0.035; Figure 2D) and more time in body contact, on average (x_Mediterranean_ = 17.3%, x_Western_ = 7.3%; ANCOVA F _[1,35]_ = 5.72, *p_HB_* = 0.055), than the Western group.

### 3.4. Diet-Induced Behavioral Changes Were Persistent

There was a significant interaction between diet and month of the treatment phase (F _[10.3,341.2]_ = 2.2, *p* = 0.019). To further understand this interaction, we compared the DAB scores and the five significantly altered behaviors in each month. The behavioral changes that we observed persisted throughout the course of the experimental diet manipulation. Holm-Bonferroni-adjusted post-hoc corrections of monthly DAB scores revealed significant diet differences in 19 out of the 24 months of behavior observation at *p* < 0.05 and an additional 4/24 months at *p* < 0.10 (Figure 1 and Supplementary Table S3 for monthly pairwise comparisons). These results indicate that the effect of diet on behavior occurred early and persisted over the two-year study.

Continuing with Holm–Bonferroni-adjusted post-hoc corrections at *p* < 0.05, the difference between diet groups in time that monkeys spent in body contact were significant at all 24 monthly timepoints of the observation period (Figure 2D). The two diet groups differed significantly in time spent alone starting in month 4 of observation (after six months of experimental diet consumption) and remained significantly different for 11 months of the treatment phase (Figure 2D). In contrast, differences in the other behaviors emerged later and were more variable over time. Time spent resting significantly differed between the Mediterranean- and Western-fed monkeys at months 9, 13, 15, and 22. The Western diet group showed significantly more anxiety behaviors at months 11 and 14. The amount of attentive time significantly differed between diet groups in months 10, 11, and 13 (Figure 2D). At a nominal threshold of uncorrected *p* < 0.05, additional months showed differences between the two diet groups in each of the behaviors described. While these months were not significant after correcting for multiple hypothesis testing, they nonetheless contribute to our understanding of the behavioral changes driven by diet (uncorrected *p* < 0.05; *n* = 24 months for the time in body contact, *n* = 19 months for time alone, *n* = 16 months for time resting, *n* = 7 months for anxiety behavior, and *n* = 10 months for time attentive; indicated by the faded triangles in Figure 2D).

### 3.5. Social Status Was Associated with Differences in Fearful Scanning, Aggression, and Submission

We tested the relationship between social status and behavior during the baseline phase of the experiment, during which the monkeys consumed a standard “monkey chow” diet. Of the twenty individual behaviors measured, seven were significantly different between the dominant and subordinate monkeys. Dominant monkeys displayed higher rates of mild (x_dominant_ = 9.5 events/h, x_subordinate_ = 2.5 events/h; t_(25.8)_ = 3.24, FDR = 0.012) and extreme (x_dominant_ = 2.3 events/h, x_subordinate_ = 0.4 events/h; t_(28.7)_ = 3.11, FDR = 0.012) aggression than subordinates. Dominant monkeys also received more mild (x_dominant_ = 9.4 events/h, x_subordinate_ = 1.3 events/h; t_(21.8)_ = 4.16, FDR = 0.003) and extreme submissions (x_dominant_ = 2.9 events/h, x_subordinate_ = 0.5 events/h; t_(26.4)_ = 3.46, FDR = 0.009). Subordinate monkeys spent more time fearfully scanning their environment (x_dominant_ = 0.028% of time, x_subordinate_ = 0.34% of time; t_(17.0)_ = −3.38, FDR = 0.012), showed more mild submission (x_dominant_ = 1.3 events/h, x_subordinate_ = 8.7 events/h; t_(21.7)_ = −4.70, FDR = 0.002), and received more mild aggression (x_dominant_ = 0.9 events/h, x_subordinate_ = 5.5 events/h; t_(22.2)_ = −4.56, FDR = 0.002) than dominant monkeys. These relationships between behavior and status observed in the baseline continued through the treatment phase, as previously reported [37].

### 3.6. Social Status Altered the Effect of Diet on Anxiety Behavior

We next examined if social status altered the effects of diet on socioemotional behaviors. During the baseline phase of the experiment, subordinate monkeys had higher levels of anxiety than dominants, but this was not a significant difference (x_dominant_ = 32.8 events/h, x_subordinate_ = 37.6 events/h; t_(21.4)_ = 1.0, *p* = 0.33). On average, anxiety declined between the baseline and experimental phases, likely reflecting habituation to a novel environment (x_baseline_ = 34.8 events/h, x_experimental_ = 29.0 events/h; t_(68.9)_ = 2.17, *p* = 0.039). However, the decline between the baseline and experimental phases was greatest in subordinates in the Mediterranean group, such that anxiety in the Mediterranean group subordinates was reduced to the level of their dominant counterparts in the experimental phase (x_dominant_ = 25.7 events/h, x_subordinate_ = 25.0 events/h). Within subordinate animals, the Mediterranean group decreased anxiety from baseline to the treatment phase significantly more than the Western group, which actually increased anxiety over the course of the experiment (x_Mediterranean_ = 28% reduction from baseline, x_Western_ = 11% increase over baseline; t_(14.9)_ = 2.37, *p* = 0.032; Figure 2E). These results suggest that diet’s effects on anxiety may depend on social status.

### 3.7. Mediterranean Diet Drove Most Behavioral Differences between Diets

**Figure 3:**
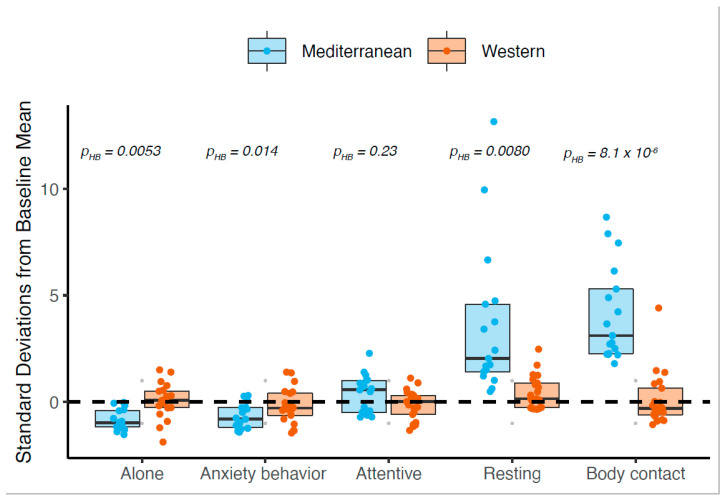
Diet-driven changes in behavior are due to changes in Mediterranean-fed animals from baseline. Western-fed animals do not show a significant difference between the baseline (time 0) and treatment phases (average of months 1–24) in any of the five behaviors that are affected by diet (all Holm–Bonferroni-adjusted *p* > 0.05). Mediterranean-fed animals show significant differences between the baseline and treatment phase in all diet-altered behaviors except the percent of attentive time. Reported Holm–Bonferroni-adjusted p (*p_HB_*) are for ANOVA of the Mediterranean group animals compared to their own baseline measurements. Blue (Mediterranean) and orange (Western) points indicate treatment phase values standardized against the across-group baseline mean (when all animals consumed a chow diet).

To understand which experimental diet may be driving changes in behavior, we examined more closely the five behaviors that showed a significant difference between diet groups. Within each diet group, we conducted one-way repeated measures ANOVA between the baseline and treatment phases of the study. There was no significant difference between the baseline and treatment phase rates of behavior in the Western group in any of the five behaviors (all Holm–Bonferroni-adjusted *p* > 0.05). In contrast, there were significant differences between the baseline and treatment phases in the Mediterranean group in time spent in body contact (F _[1,16]_ = 60.4, Holm–Bonferroni-adjusted p (*p_HB_*) = 8.1 × 10^−6^), time spent alone (F _[1,16]_ = 18.2, *p_HB_* = 0.0053), time spent resting (F _[1,16]_ = 15.3, *p_HB_* = 0.0080), and rate of anxiety behavior (F _[1,16]_ = 13.4, *p_HB_* = 0.014), but not attentive time (F _[1,16]_ = 4.7, *p_HB_* = 0.23). These observations suggest that most of the observed differences between diet groups in the treatment phase were due to changes in behavior of those in the Mediterranean group (Figure 3).

## 4. Discussion

In this randomized preclinical trial in nonhuman primates, we found that two common human diet patterns had profoundly different effects on physiological and behavioral outcomes [35,36,37,38,39]. This study expands on findings from a prior report, which showed that diet drove inflammatory polarization of circulating immune cells and altered behavior in ways that had both social (Mediterranean diet-fed animals were more socially integrated) and psychological (Mediterranean diet-fed animals exhibited fewer anxiety behaviors) consequences, and that behavioral changes were associated with some of the diet effects on gene expression [39]. Here, we delved more deeply into the character of the diet-induced behavioral changes and found that the experimental diets rapidly and persistently shift affiliation, activity, and anxiety in cynomolgus macaques and differently impact socially dominant and subordinate individuals.

The Mediterranean diet increased the time spent in body contact and decreased the time spent alone, indicating more time spent in affiliation. The diet-driven changes in affiliation were observed in the first month of behavior observations (the third month on the experimental diets) and persisted throughout the course of the study. Affiliation reflects a monkey’s social integration or isolation, a key component of the social environment impacting health outcomes. In humans, social isolation has been associated with inflammation [57,58,59] and is a risk factor for psychiatric disorders [18,60,61]. Thus, given the physiological and behavioral similarities shared between humans and NHPs [31], the higher isolation in Western diet-fed monkeys relative to Mediterranean diet-fed monkeys supports the link between components of the Western diet and psychiatric disorders. Reduced social interaction has been observed in rodents fed a Western-style diet [62,63] and in the offspring of rodents [64,65,66] and female Japanese macaques [40] that consumed a Western-style diet during gestation. In human observational studies of self-reported diet and social characteristics, social isolation is associated with Western diet consumption [41,67], whereas adherence to a Mediterranean diet is correlated with decreased isolation [68]. The findings reported here are novel in their observations, including the rapidity with which behavior changed in response to dietary changes; the longitudinal persistence of behavior changes over two years (a timespan roughly equivalent to seven human years); the observation that the Mediterranean diet drove most of the differences in behavior and the randomized trial design that allows for causal inferences. These findings suggest that dietary intervention may have a clinical application in the long-term treatment of adverse psychosocial effects of social isolation.

Another psychologically relevant behavioral change is that the Mediterranean diet decreased anxiety-like behaviors, resulting in lower levels of anxiety than in Western diet-fed animals. This main effect of diet was largely due to an elevated rate of anxiety behaviors in subordinate animals, which was ameliorated by the dietary intervention in Mediterranean group subordinates. All diet-by-status groups except the socially subordinate Western animals exhibited average decreases in anxiety behaviors over time. In fact, Mediterranean group subordinates reduced anxiety behaviors to the level of their dominant counterparts (x_dominant_ = 25.7 events/h, x_subordinate_ = 25.0 events/h). This observation suggests that the Mediterranean diet could be an effective intervention for anxiety. Likewise, it may be that anxiolytics would be more efficacious in the Mediterranean than in the Western diet background—a hypothesis that needs testing.

Most of the diet-induced behavioral changes were driven by changes in the behavior of the Mediterranean group monkeys. Behaviors of the Western group monkeys did not change significantly from baseline. This implies that the Western diet resembles standard lab chow, at least with respect to effects on behavior and, by extension, central nervous system function (see Table 1). From this perspective, it is notable that the NHP Western diet and chow are similar in omega-6:omega-3 fatty acid ratios. Indeed, circulating omega-6:omega-3 fatty acid ratios are positively associated with anxiety and depression in humans [69]. The data reported here suggest that those associations may be causal. Likewise, changes in behavior in the Mediterranean group may be due to characteristics unique to that diet. In support of this, data from both humans and model organisms indicate a preventative role for omega-3 and polyunsaturated fatty acids in anxiety disorders [70,71]. Notably, there were no diet effects on aggressive or submissive behaviors sent or received.

The observed changes in behavior are suggestive of underlying physiological changes that may have important health consequences. There are multiple pathways through which diet may alter behavior—mediated or initiated through the CNS—some of which have already been supported by previous findings from this study. We previously demonstrated that, relative to the Mediterranean group, the Western group had reduced gut microbiota diversity [36], a well-established modulator of CNS activity through the gut-brain axis [72]. The Western group exhibited increased caloric intake, body fat, insulin resistance, and sympathetic nervous system and hypothalamic–pituitary–adrenal activity relative to the Mediterranean group [35,37]—all of which have been associated with either social isolation or anxiety or both [73,74,75,76,77,78]. We also demonstrated that the Western group had a proinflammatory monocyte transcriptome [39], consistent with numerous findings supporting links between the Western diet and inflammation [79,80,81,82,83] versus reduced inflammation with the Mediterranean diet and its components [84,85,86]. The peripheral inflammatory milieu can alter CNS activation through modulation of the vagal activity or direct infiltration by cytokines passing the blood-brain barrier [87,88,89]. Likewise, inflammatory cytokine production is sensitive to social isolation [18,90,91]. Consistent with these plausible mechanisms, we previously reported that diet-altered behavior may mediate, or be mediated by, some proinflammatory shifts in immune cell gene expression [39].

In addition to lasting negative psychosocial effects, social isolation and inflammation are associated with worse infection outcomes. For example, in the ongoing COVID-19 global pandemic, patterns of differential mortality by country and region are associated with dietary patterns variations [92,93,94]. Lower mortality rates from COVID-19 are observed in countries/regions that consume higher levels of antioxidants and seafood and lower levels of sugar and animal products [93,95], and omega-3 fatty acid supplementation was associated with reduced mortality and improved respiratory and renal function in a randomized clinical trial [96]. Thus, Mediterranean diet consumption may ameliorate infection severity via reduced levels of inflammation. Taken together, diet composition may impact infectious disease progression—exacerbation by the Western diet or amelioration by the Mediterranean diet—through both behavioral and inflammatory pathways. Thus, dietary intervention may mitigate some of the deleterious health effects of social isolation and inflammation in COVID-19 and perhaps other infectious diseases [97,98].

In summary, the data presented here and in our previous report from the same NHP preclinical trial strongly support the hypothesis that a Mediterranean-like diet beneficially influences health through behavioral as well as physiological pathways and may be a critical component of a healthy lifestyle that increases positive social interactions while reducing anxiety and the risk of multiple chronic diseases of aging. Future studies should assess if Mediterranean diet consumption in conjunction with other therapeutic interventions is more efficacious than standard therapies alone for treating anxiety and inflammatory diseases.

## Figures and Tables

**Table 1:** Comparison of Nonhuman Primate Diet Compositions Used in the Current Study with Human Diet Patterns.

Diet Composition	Human		Nonhuman Primate		
	Western	Mediterranean	Western ^1^	Mediterranean ^1^	Chow ^2^
% of Calories					
Protein	15 [42]	17 [43]	16	16	18
Carbohydrate ^3^	51 [42]	51 [43]	54	54	69
Fat	33 [42]	32 [43]	31	31	13
% of Total Fats					
Saturated	33 [42]	21 [43]	36	21	26
Monounsaturated	36 [42]	56 [43]	36	57	28
Polyunsaturated	24 [42]	15 [43]	26	20	32
Other Nutrients					
ω6:ω3 Fatty Acids	15:1 [44]	2.1–3:1 [45]	14.8:1	2.9:1	12:01
Cholesterol mg/Cal	0.13 [42]	0.16 [43]	0.16	0.15	trace
Fiber g/Cal	0.01 [42]	0.03 [46]	0.02	0.04	0.01
Sodium mg/Cal	1.7 [42,47]	1.3 [43,46]	1.7	1.1	0.25

^1^ Developed and prepared at Wake Forest School of Medicine [35]. ^2^ LabDiet Chemical Composition Diet 5037/8. Type of fat known in 86% of total fat. Omega-6 from corn and pork fat; ^3^ Human carbohydrate calories include alcohol. Reprinted with permission [35].

## Data Availability

The data presented in this study are available upon request from the corresponding author.

## Supplementary Materials

The following supporting information can be downloaded at: https://www.mdpi.com/article/10.3390/nu14142852/s1. Table S1. Experimental Diet Compositions; Table S2. Dependent Variables Measured or Calculated in Behavioral Characterization of Diet Trial; Table S3. Mediterranean vs. Western Group DAB Scores by Month.
