# Supplementary material for: Mediterranean Diet Reduces Social Isolation and Anxiety in Adult Female Nonhuman Primates
Source: Nutrients. 2022 Jul 12;14(14):2852. doi: 10.3390/nu14142852 (PMC9322105; doi:10.3390/nu14142852)
Supplement: Supplementary file 1 [file nutrients-14-02852-s001.zip › nutrients-1766802-supplementary.pdf]

Supplementary Table S1. Experimental Diet Compositions.

Western-like Diet		Mediterranean-like Diet	
**Ingredient**	**g/ kg**	**Ingredient**	**g/kg**
Casein, USP	85.0	Casein, USP	17.4
Whey Protein - 895 895	85.0	Whey protein - 895	17.4
		Dried Egg white	26.1
		Fishmeal (Menhaden)	26.1
		Walnuts	8.7
		Black Bean flour	43.5
		Garbanzo Bean flour	17.4
		Wheat Flour (all purpose)	243.5
Dextrin	260.0	Dextrin	96.6
Sucrose	180.0	Sucrose	34.8
High Fructose Corn Syrup 55	70.0	Banana	1304
		Applesauce	38.2
		Tomato paste	17.4
Cellulose (Alphacel)[1]	79.4	Cellulose (Alphacel) [1]	94.8
Lard	41.5	Olive Oil (Filippo Berio Extra Virgin)	61.7
Beef Tallow HHR	40.0	Menhaden Oil (Omegapure)	8.7
Butter, lightly salted	12.5	Butter, lightly salted	8.7
Corn Oil	35.0	Corn Oil	10.4
Flaxseed oil	3.0	Flaxseed oil	1.7
Dried Egg Yolk	6.0	Dried Egg Yolk	14.8
Crystalline Cholesterol	0.4		
Complete Vitamin Mix (Teklad)	25.0	Complete Vitamin Mix (Teklad)	21.7
Mineral Mix w/o Ca, P, NaCl	50.0	Mineral Mix w/o Ca, P, NaCl	43.5
Calcium Carbonate	4.3	Calcium Carbonate	3.7
Calcium Phosphate, Monobasic	7.5	Calcium Phosphate, Monobasic	6.5
NaCl (Table Salt)	16.0	NaCl (Table Salt)	6.3
TOTAL	1000	TOTAL	1000

[1] Total Fiber (% of diet): WEST: 7.94; MED: 12.7

Supplementary Table S2. Dependent Variables Measured or Calculated in Behavioral Characterization of Diet Trial.

Dependent Variable	Operational Definition
Affiliation Behaviors Observed	
Percent of Time Spent Alone	Percent of time spent out of monkey's arm's reach of conspecifics
Percent of Time Spent in Body Contact	Percent of time spent in contact with conspecific(s)
Percent of Time Spent in Close Proximity	Percent of time spent within monkey's arm's reach of conspecific(s)
Activity Behaviors Observed	
Percent of Time Spent Attentive	Percent of time monkey alert and attending to an object or event
Percent of Time Spent Fearfully Scanning	Percent of time visual scanning of the environment
Percent of Time Spent in Locomotion	Percent of time walking or running along the ground or over suspended surfaces (> 1 m/min)
Percent of Time Spent Investigating	Percent of time exploratory behavior directed at environment
Percent of Time Spent Lying Down	Percent of time lying down with eyes open
Percent of Time Spent Resting	Percent of time resting with eyes closed or asleep
Anxiety Behavior Observed	
Rate of Anxiety Behavior	Frequency of anxiety-related behaviors, including scratching, itching, and grooming self
Social Behaviors Observed	
Percent of Time Spent Being Groomed	Percent of time being groomed by conspecific(s)
Percent of Time Spent Grooming	Percent of time grooming conspecific(s)
Rate of Extreme Agression	Frequency of extreme aggressive behaviors including bite, slap, grab, chase, charge, and lunge
Rate of Extreme Agression Received	Frequency of receiving extreme aggressive behaviors including bite, slap, grab, chase, charge, and lunge
Rate of Extreme Submission	Frequency of extreme submissive behaviors including scream, squeal, scream threat, crouch, and flee
Rate of Extreme Submission Received	Frequency of receiving extreme submissive behaviors including scream, squeal, scream threat, crouch, and flee
Rate of Mild Aggression	Frequency of mild aggressive behaviors including display, displace, and threat (open mouth, stare, or yawn)
Rate of Mild Aggression Received	Frequency of receiving mild aggressive behaviors including displace and threat (open mouth, stare, or yawn)
Rate of Mild Submission	Frequency of mild submissive behhaviors including lip smack, grimace, submissive present, crouch and move away
Rate of Mild Submission Received	Frequency of receiving mild submissive behhaviors including lip smack, grimace, submissive present, crouch and move away
Composite Behavior Calculated	
Diet-Altered Behavior (DAB) Score	Linear combination of all 20 observed behaviors, scaled and multiplied by the loading of each variable onto PC2 (from Johnson et al., 2021) and weighted by its eigenvalue

Supplementary Table S3. Mediterranean vs. Western Group DAB Scores by Month.

Month of Observation (months on diet)	Mediterranean Group *Mean (S.D.)*	Western Group *Mean (S.D.)*	Statistical Analysis	Test Statistic	*p-value*	*Holm-Bonferroni adjusted p-value*
Baseline	-0.09 (1.4)	0.07 (1.6)	t-test (df = 36.0)	-0.3	0.73	-
1 (3)	0.54 (1.9)	-0.43 (1.5)	ANCOVA (df = [1, 35])	27.2	**0.014***	0.070
2 (4)	0.56 (2.0)	-0.45 (1.7)	ANCOVA (df = [1, 35])	29.1	0.016	0.070
3 (5)	0.54 (1.9)	-0.44 (1.6)	ANCOVA (df = [1, 35])	27.8	0.015	0.070
4 (6)	1.09 (1.7)	-0.88 (1.4)	ANCOVA (df = [1, 35])	15.9	1.8×10^{-5}	$\mathbf{3.0 \times 10^{-4}}$
5 (7)	1.18 (1.8)	-0.95 (1.6)	ANCOVA (df = [1, 35])	17.4	2.4×10^{-5}	$\mathbf{3.6 \times 10^{-4}}$
6 (8)	1.03 (1.9)	-0.83 (1.6)	ANCOVA (df = [1, 35])	14.7	2.0×10^{-4}	**0.0024**
7 (9)	0.89 (1.6)	-0.72 (1.8)	ANCOVA (df = [1, 35])	60.7	1.3×10^{-5}	$\mathbf{2.3 \times 10^{-4}}$
8 (10)	1.23 (1.6)	-1.00 (1.4)	ANCOVA (df = [1, 35])	10.6	4.3×10^{-6}	$\mathbf{8.6 \times 10^{-5}}$
9 (11)	1.17 (1.9)	-0.95 (1.4)	ANCOVA (df = [1, 35])	12.6	2.9×10^{-5}	$\mathbf{4.0 \times 10^{-4}}$
10 (12)	1.22 (1.5)	-0.99 (1.4)	ANCOVA (df = [1, 35])	4.4	1.8×10^{-5}	$\mathbf{3.0 \times 10^{-4}}$
11 (13)	1.26 (1.1)	-1.02 (1.5)	ANOVA (df = [1, 36])	26.4	9.9×10^{-6}	$\mathbf{1.9 \times 10^{-4}}$
12 (14)	1.01 (1.6)	-0.82 (1.4)	ANCOVA (df = [1, 35])	4.9	2.9×10^{-4}	**0.0032**
13 (15)	1.36 (2.1)	-1.10 (0.9)	ANCOVA (df = [1, 35])	10.0	2.6×10^{-6}	$\mathbf{5.4 \times 10^{-5}}$
14 (16)	1.66 (1.3)	-1.34 (1.7)	ANCOVA (df = [1, 35])	19.4	6.2×10^{-9}	$\mathbf{1.5 \times 10^{-7}}$
15 (17)	1.55 (1.4)	-1.26 (1.6)	ANCOVA (df = [1, 35])	7.0	2.4×10^{-7}	$\mathbf{5.4 \times 10^{-6}}$
16 (18)	0.68 (1.3)	-0.55 (1.5)	ANCOVA (df = [1, 35])	11.8	0.0020	**0.016**
17 (19)	1.20 (1.5)	-0.97 (1.3)	ANCOVA (df = [1, 35])	19.4	3.6×10^{-7}	$\mathbf{8.0 \times 10^{-6}}$
18 (20)	0.97 (1.8)	-0.78 (1.5)	ANCOVA (df = [1, 35])	8.9	6.1×10^{-4}	**0.0061**
19 (21)	1.20 (1.8)	-0.84 (1.5)	ANCOVA (df = [1, 35])	14.6	7.8×10^{-5}	**0.0010**
20 (22)	0.30 (2.1)	-0.24 (1.4)	ANCOVA (df = [1, 35])	10.0	0.23	0.23
21 (23)	0.86 (2.0)	-0.70 (1.6)	ANCOVA (df = [1, 35])	12.7	0.0020	**0.016**
22 (24)	0.70 (1.9)	-0.56 (1.2)	ANCOVA (df = [1, 35])	15.4	0.0030	**0.018**
23 (25)	0.54 (1.9)	-0.44 (1.3)	ANCOVA (df = [1, 35])	7.6	0.032	0.070
24 (26)	0.86 (1.8)	-0.70 (1.5)	ANCOVA (df = [1, 35])	15.9	7.4×10^{-4}	**0.0066**

**A priori* hypothesis, thus reported p-value was not corrected for multiple hypothesis testing.

Supplementary Note S1: **Diet Preparation**. Ten-kilogram batches of the experimental diets were prepared at a time. Batches were stored frozen at -20°C until thawing. Daily portions were thawed in the refrigerator prior to feeding. To prevent oxidation of lipids and polyphenols, extra-virgin olive oil was stored under argon. To ensure stability of each of the experimental diets, samples from three batches – one early, one midway, and one near study's end - were assayed for macronutrient and fatty acid content.
